# Radiographic Evaluation of Root Canal Treatment Performed by Undergraduate Students, Part I; Iatrogenic Errors 

**DOI:** 10.22037/iej.v13i1.16800

**Published:** 2018

**Authors:** Patrícia Zambon da Silva, Francisco Carlos Ribeiro, Juliana Machado Barroso Xavier, Rodrigo Pratte-Santos, Cristina Demuner

**Affiliations:** a *Federal University of Espírito Santo, Vitória, ES, Brazil; *; b * Department of Dental Clinic, Dental School, Federal University of Espírito Santo, Vitória, ES, Brazil; *; c * Department of Dental Clinic, Dental School, Federal University of Espírito Santo, Vitória, ES, Brazil; *; d *Department of Pathology, Health Sciences Center, Federal University of Espírito Santo, Vitória, ES, Brazil;*; e * UFES, Federal University of Espírito Santo, Vitória, ES, Brazil*

**Keywords:** Dental Student, Endodontics, Errors, Iatrogenic, Radiography, Root Canal Therapy

## Abstract

**Introduction:**

The root canal preparation is an important stage in the undergraduate teaching and must be handled with care. Iatrogenic mishaps may occur during this procedure which might compromise the success of endodontic treatment. The aim of this study was to determine, the frequency of iatrogenic errors in endodontic treatments provided by undergraduate dental students at the School of Dentistry of Federal University of Espírito Santo (UFES), Brazil.

**Methods and Materials:**

Radiographic records of 511 anterior teeth and pre-molars with endodontic treatment performed by undergraduate students, between 2012 and 2014 were randomly chosen. The final sample consisted of radiographic records of 397 teeth endodontically treated and were evaluated by using the projection of radiographic images. Iatrogenic errors that were detected in root filled teeth included: apical perforation, root perforation, furcation perforation, strip perforation, presence of fractured instruments, ledge and zip. Then they were classified, according to the absence or presence of iatrogenic errors, as adequate or inadequate.

**Results:**

According to the results, 7.3% of the teeth were inadequate, and there was no statistically significant difference among the groups of anterior teeth, incisors, or canines (*P*>0.05). A ledge was present in 6.54% of root canals, a zip in 0.75% of root canals, and only one root canal presented a fractured instrument. In teeth with moderate curvature, the root curvature was a factor that possibly influenced the occurrence of the ledge (*P*<0.05).

**Conclusion:**

The majority of root canal preparations showed a low occurrence of iatrogenic errors.

## Introduction

The European Endodontic Society, in elaborating the Undergraduate Endodontic Curricular Guidelines, defined that this specialty is a ramification of dental science. The objective is studying the form, function and health of tooth pulp and the periradicular region, and their respective treatments and follow-up [1-3].

The treatment of the root canal system is constituted of various stages, which in an interdependent form, possesses a high capacity to influence the final result [4]. Therefore, the progressive conical modeling of the root canal becomes fundamental in order to perform the enlargement of the medium and cervical thirds, and preserve the smallest possible root apex diameter, maintaining its original trajectory and allowing the obturation of the root canal in a hermetic and three-dimensional manner [5].

The procedural errors which occurred during the instrumentation phase, such as the formation of ledges, the root perforations and the instrument fractures, among others, could hinder the conclusion of the intracanal procedures in an appropriate manner, and consequently, compromise the success of treatment. There is high potential for failure of the endodontic treatment when a procedure error occurs during its execution, principally in necrotic teeth, in view of its incapacity of eliminating the dentinal remains and infected debris [6, 7].

The literature demonstrates that the percentage of most endodontic treatments that are considered acceptable, yet are still inferior to the ideal, varying from approximately 26 to 55% [8-10]. These low rates are also observed, when the endodontic treatments were executed by undergraduate students are evaluated, expressing values from 10.9% to 55.3% [11-21]. In this context, the teaching and learning process could be questioned as to if it was insufficient for the graduating student, not focusing on the principles and objectives in all stages of endodontic treatment [22].

Few studies emphasize the detection of iatrogenic errors (apical perforation, root perforation, furcation perforation, strip perforation, presence of fractured instruments, ledge, and zip) during the preparation of the root canal [11, 12, 15, 16]. However, it is important to highlight that the procedural errors could compromise the cleansing and preparation of the canal, resulting in an incomplete obturation, which could compromise the success of the treatment [23]. The undergraduate Curriculum Guidelines for Endodontology, in accordance with the European Endodontic Society, determine that students must be capable of identifying and avoiding common procedural errors during the instrumentation of root canals, including ledges, fractured instruments and perforated roots [2, 3].

The aim of this study was to identify the presence of iatrogenic errors which occured in endodontic treatments performed by undergraduate students in the Dental School of the Federal University of Espírito Santo (UFES), Brazil. And also to evaluate if the curvature of the root canal had an influence on the frequency of the most common iatrogenic error detected.

## Materials and Methods

Records of 511 endodontic tooth treatments, performed by undergraduate students of the third-year of the Endodonic II discipline at UFES, Brasil, during the period from 2012 to 2014, were collected randomly.

The inclusion criteria were the complete radiographic records of the teeth including initial, transoperatives (working length and master cone test) and the final radiograph of each endodontic treatment, with good radiographic technique or processing. It should be highlighted that only endodontic treatments of the incisor, canine, and premolar tooth-type were included in this study. The exclusion criteria were incomplete radiographic records, poor technical radiographic or inadequate processing, non-dissociation of filled root canals in multirooted teeth, and the overlapping of anotomic structures in the root canals. Root resorptions, calcifications, retreatments and teeth with incomplete root formation were also excluded.

The endodontic treatments performed by undergratuated students followed a standardized and aseptic technique, with absolute isolation in all treatments, following the guidelines suggested by the Endodontic II discipline. The working length was determined with the use of radiographs. All teeth were instrumented with the modified Oregon technique [24] using stainless steel K-files (Dentsply Maillefer, Ballaigues, Switzerland) of 0.02 taper and K-Flexofile (Dentsply Maillefer, Ballaigues, Switzerland) of 0.02 taper flexible instruments that were used on the curved root canals. Gates-Glidden drills (Dentsply Maillefer, Ballaigues, Switzerland) were used in the coronal third of the root canal in order to facilitate a straight-line access to the root apical third. Root canals were irrigated with 2.5% sodium hypochlorite in cases of necropulpectomy, and 1% in cases of biopulpectomy. All canals were filled with gutta-percha cones (Odous de Deus, Belo Horizonte, Brazil) and AH-Plus (Dentsply Maillefer, Ballaigues, Switzerland) endodontic cement using Tagger's hybrid technique [25].

The strict criteria for the detection of iatrogenic errors in radiographic records were defined by three examiners, independent endodontists by discussing its application in some cases. The examiners were calibrated in two stages. The first stage, determined the inter-examiner agreement, by evaluating 50 radiographic records of randomly selected teeth that had been treated endodontically. In the second stage, the inter-examiner calibration occured one month after the first evaluation, and the same 50 radiographic records were reevaluated by the three examiners [11].

The iatrogenic errors evaluation was based on radiographic records of endodontic treatments which included the initial, transoperatives (working length and master cone test) and the conclusion radiograph of each treatment. The visualization of the periapical radiographies were standardized and performed by their projection using a slide projector (Kodak-Ektagraphic III, São Paulo, SP, Brazil) on a white background in a dark environment, amplified to a 1:10 scale.

The radiographic detection criteria for the presence of iatrogenic errors in root canals filled were:

- Apical perforation: identified when the apical termination of the filled canal was different from the original canal terminus, or when the filling material was extruding through the apical foramen [15].
- Root perforation: identified when the extrusion of filling material was detected in any other area of the root, except the furcation area and the lateral (inner) wall of the root [11, 15].
- Furcation perforation: identified when the extrusion of fillling material through the furcation area, was detected in multirooted teeth [11].
- Fractured instrument: identified through observation of the radiograph and according to the radiopacity between the filling material and the fractured instrument [16].
- Ledge: identified when the root filling was at least 1 mm shorter than the initial working length, or deviated from the original curvature canal shape [11, 15].
- Zipping: identified when the apical termination of the filled canal appeared as an elliptical shape transported to the outer wall [11].
- Strip perforation: identified when the extrusion of the material was detected on the lateral (interior) wall of the root of any tooth [11, 15].

The presence of iatrogenic errors on radiographies, specifically in root canals filled, were evaluated and classified in an independent form in relation to the presence or absence of iatrogenic errors. The classification of teeth corresponded to the filled root canal with the worst classification, since the tooth was considered as a unit. Thus, the tooth that presented the absence of iatrogenic errors in the root canals was classified, according to the chemical-mechanical preparation, as adequate. Consequently, teeth that presented iatrogenic errors were classified as inadequate.

The clinical parameters as tooth-type and root curvature were also considered for the detection of iatrogenic errors in the root canals filled. The root curvature was determined and classified in accordance with Schneider: a curvature<5^°^: tooth considered straight, curvature<20^°^: moderate, and curvature≥25^°^: severe [26]. 

Data were inserted in the Excel softwere and the statistical tests were performed using the SPSS software for Windows (SPSS version 17.0, SPSS Inc., Chigago, IL, USA). The examiners agreement was measured by Cohen kappa test, with assistance of the MedCalc program, version 15.2.2. The *chi* square test for proportions was used in the comparisons between the percentages.

## Results

The k-values obtained for the inter-examiner agreement was 0.80 and 0.90 for the intra-examiner agreement, indicating an ideal agreement between the examiners. 

A total of 114 radiographic records of endodontically treated teeth were excluded. Among them, 16 were radiographic errors, 61 incomplete records, 31 retreatments, 4 incomplete root formations, and 2 teeth with resoprtion in the root canals walls. Thus, the final sample constituted 397 radiographic records of endodontically treated teeth, with a total of 480 filled root canals. The sample was constituted of radiographic records of 397 endodontically treated teeth, corresponding 480 roots canals filled. The distribution of this sample according tooth-type is detailed in Table 1. The most prevalent teeth were premolars 219 (55.2%), followed by 132 (33.2%) incisors and 46 (11.6%) canines.

The frequency of endodontically treated teeth defined as “inadequate” were found in 29 (7.3%) teeth, while 368 (92.7%) were considered as “adequate” (Table 2). There was no significant difference (*P*>0,05) among the tooth-type of incisors, canines and premolars.

Table 3 represents the percentual distribution of each type of iatrogenic errors detected in root canals filled according to the tooth-type. Ledge formation were found in 26 (6,54%) root-canals from wich were 5 (3,8%) incisors, 3 (6,5%) pre-molas and 18 (8,2%). Zip was observed 3 (0.75%) root canals and was only detected in pre-molars. Fractured instrument was observed only in 1 root canal . The frequency of iatrogenic errors detected was not significantly different between the root canals filled evaluated according tooth-type of incisors, canines and pre molars. 

**Table 1:** Distribution of tooth-type and root canals treated

**Tooth type**	**Teeth ** **N(%)**	**Root canals N(%)**
**Incisors **	132 (33.2)	132 (27.5)
**Canines **	46 (11.6)	46 (9.6)
**Premolars **	219 (55.2)	302 (62.9)
**Total**	397 (100.0)	480 (100.0)

**Table 2:** Distribution of adequate and inadequate teeth performed by students according to tooth-type

**Tooth type**	**Adequate**	**Inadequate**	***P*** **-value**
	**N (%)**	**N (%)**	
**Incisors **	6 (20.7)	126 (34.2)	0.200
**Canines **	3 (10.3)	43 (11.7)	0.941
**Premolars **	20 (69.0)	199 (54.1)	0.174
**Total**	29 (100)	368 (100)	

In respect to canal curvature, 375 (78,1%) root canals were straights, 92 (19,2%) had moderate curvatures and 13 (2,7%) with severe curvatures. The ledge in all teeth was found in 14 (3.7%) root canals fillled classified with straight curvatures, 11 (11.9%) root canals filled with moderate curvatures, and 1 (7.7%) root canal fillled with severe curvature. A significant statistical difference was verified between moderate and straight and curved root canals (*P*<0,05) (Table 4).

## Discussion

The evaluation of iatrogenic errors was based on the image of root canal filled on the peripical radiograph of cases treated by undergraduated students. The method using periapical radiographs constitute the most commonly employed method in the evaluation of endodontic treatments performed by undergratuated students of teaching institutions, which is in accordance with others studies in the literature [11,12, 14-19,26]. However, the radiographic evaluation is a limiting feature because they provide two-dimensional images of three-dimensional objects [19].

The iatrogenic errors were detected in 30 root canals filled; as a consequence 29 (7.3%) teeth were classified as inadequate. This frequency was close to those reported by Vukadinov *et al.* [19] (3.4%) of the filled root canals and Kulic *et al.* [17] where 7 teeth were detected with iatrogenic errors. Also, Lynch and Burke [27] did not detect the presence of iatrogenic errors and Rafeek *et al.* [27] identified fractured instruments in 1.5% of the root canals. In contrast, Yousuf, Khan and Mehdi [21] identified iatrogenic errors in 32.8% of teeth, and Haji-Hassani *et al.* (20) in 66% of teeth.

In the present study, the percentage of iatrogenic errors was evaluated according tooth-type and no significant difference was found among them. However, several studies detected differences between the frequency of iatrogenic errors and the tooth-type evaluated [13, 15, 16]. 

On the other hand, the inclusion of the molar tooth group could be a factor of influence in the presence of iatrogenesis, on the basis that they possess a more complex anatomy and higher degree of curvature [11, 13, 15, 16]. In the face of this argumentation, it is suggested that the non-inclusion of this variable, in the present study and others [17, 18, 27], possibly favored the low frequency of iatrogenic errors.

The most frequent iatrogenic errors detected in the sample were ledges in 6.54% of root filled canals. This frequency was close to those reported by Vukadinov *et al. *[19] 2.8% and lower to others; Khabbaz *et al.* [16] 54.8% and Mozayeni *et al.* [13] 26%.

The analysis of canal curvature related to the presence of the ledge indicated that moderate curvature compared to straight canals showed evidence of significant statistical difference between the percentages. Canal curvature was a factor related to the presence of ledges in canals with moderate curvature. Likewise, other authors affirmed that the root curvature is a factor related to the presence of ledges [11, 14]. 

The low frequency of iatrogenic errors in the present study could be attributed to the standardization of instrumentation techniques employed in the discipline. The students develop a single crown-down instrumentation technique, founded on the principle of a segmented and progressive preparation from the cervical third. This reduces the tension of the instrument when it works in the critical apical zone. In addition, there is the use of flexible instruments for canals, independent of the curvature degree. 

**Table 3:** Distribution of iatrogenic errors in root canals filled according to tooth-type

**Error**	**Tooth type N(%)**			**Total** **root** **canals**	***P*** **-** **value**
	**Incisors**	**Canines**	**Premolars**		
**Ledge**	5 (3.8)	3 (6.5)	3 (8.2)	26	Ns
**Zip**	-	-	-	3	
**Fractured instrument**	1 (0.8)	-	-	1	
**Perforated root**	-	-	-	-	
**Perforated apical**	-	-	-	-	
**Perforated furcation**	-	-	-	-	
**Strip perforation**	-	-	-	-	

Ns: There is no significant difference;

** : Test not performed due do large number of zeroed cells

**Table 4:** Percentage of ledges in all root canals filled according to canal curvature

	**Number of root canals**	**Ledge ** **of root canals N(%)**
**Straight**	375	14 (3.7^a^)
**Moderate**	92	11 (11.9^b^)
**Severe**	13	1 (7.7^a,b^)

Note: Different letters denote significant statistical differences

On the other hand, Lynch and Burke [27], Kulic *et al.* [17], Rafeek *et al.* [18], and Vukadinov *et al.* [19] also obtained low frequencies of iatrogenic errors, using the step-back technique. However, these studies presented some characteristics that possibly contributed to this fact. Among them are the use of flexible instruments, the reduction of the number of iatrogenic criteria evaluated, the absence of significant curvatures, and teeth with complex anatomies.

It is valid to emphasize that prior to each endodontic treatment, the students, as an obligatory routine, develop a detailed and illustrated treatment plan for each clinical treatment to be performed. This plan is discussed and corrected by the professors of the discipline. At the end of each concluded treatment, the students receive a feedback from the professors, in relation to the quality of the concluded treatment, which could have contributed to the low percentages of encountered iatrogenic errors.

Another factor, which could have contributed to the low frequency of iatrogenic errors in the present study, is related to the supervision of the students by professors specialized in Endodontics. In addition, the proportion of that supervision in the clinic was carried out with teacher staff to student ratio of 1:7. Similar results were obtained by Vukadinov *et al.* [19] in which the supervision of students was performed by specialists with a 1:8 teachers/students ratio. However, in studies that present highest frequencies of iatrogenic errors, It is valid to emphasize that prior to each endodontic treatment, the students, as an obligatory routine, develop a detailed and illustrated treatment plan for each clinical treatment to be performed. This plan is discussed and corrected by the professors of the discipline. At the end of each concluded treatment, the students receive a feedback from the professors, in relation to the quality of the concluded treatment, which could have contributed to the low percentages of encountered iatrogenic errors.

However, in studies that present highest frequencies of iatrogenic errors, such as Khabbaz *et al.* (19) the proportion of professors to students was 1:15, a fact that could have been attributed to the results obtained. In addition, Balto *et al.* (15) argued that the supervision of students in a 1:12 ratio is not ideal, also this study was not performed with specialists in Endodontics. 

The Curricular Guidlelines of the Endodontic II discipline of UFES have the objective of respecting the learning curve of the students, since they perform for the first time, *in vivo*, the techniques and principles of endodontic preparation learned in the endodontic preclinic themselves. Thus, simpler treatments are recommended to be performed first: the incisor, canine and premolar tooth tooth-type. Afterwards, in the sequence of the cirrucular program, the student will be prepared for more advanced treatments. In this manner, this work opens perspectives for the comparison of treatments performed by the students of advanced classes performing more complex treatments, and in this context, permitting a possible confirmation of success in the teaching-learning relationship.

As stated, numerous factors have an impact on the low percentage of iatrogenic errors detected in this study. Among them are the tooth-type of teeth evaluated, the methodology criteria, the types of endodontic instruments employed, the proportion of students to professors, and the qualification of the supervisors of the students during the clinical activities.

## Conclusion

The root canal treatment performed by undergratuate students was classified as “inadequate” in 7.3% of the cases. Canal curvature had an influence in the formation of the ledge on the canals with moderate curvature.
